# Prescription for healing the climate crisis: Insights on how to activate health professionals to advocate for climate and health solutions

**DOI:** 10.1016/j.joclim.2021.100082

**Published:** 2021-10

**Authors:** Kate T. Luong, John Kotcher, Jeni Miller, Eryn Campbell, Elissa Epel, Mona Sarfaty, Edward Maibach

**Affiliations:** a George Mason University, 4400 University Drive, MS 6A8, Fairfax, VA 22030, USA; b The Global Climate and Health Alliance, 299 Panoramic Way, Berkeley, CA 94704, USA; c University of California, San Francisco, 3333 California Street, San Francisco, CA 94118, USA

**Keywords:** Climate and health solutions, Health professional advocates, Program development

A stable climate is the most fundamental determinant of human health, making climate change arguably the greatest public health threat facing humanity today [1,2]. Large-scale, coordinated policy changes are urgently needed to adequately address this global crisis [3].

Health professionals have a unique and essential role to play as advocates for climate solutions [4]. First, they have unparalleled assets in terms of their knowledge, skills, credibility, and access to important audiences—including policy makers, government officials, educators, patients, and the general public—both in and beyond their own community [5]. Second, many health professionals are aware of and concerned about the health impacts of climate change [6] and have called for integrating climate advocacy as part of their professional responsibility [7], [8], [9], [10]. Lastly, and perhaps most importantly, two recent multi-national surveys of health professionals found that the vast majority of respondents were supportive of advocating for climate solutions [11], [12], [13].

Research conducted over the past decade on the American general public has shown that, while many people are concerned about climate change, relatively few actually take actions to support the enactment of climate policies [14]. These findings echo the results from an audience segmentation study called Global Warming's Six Americas [14,15], which found that Americans can be categorized into six distinct groups based on their beliefs and attitudes about climate change: Alarmed, Concerned, Cautious, Disengaged, Doubtful, or Dismissive; and that members of only one segment—the “Alarmed”—are willing to take such meaningful climate actions. Despite this high degree of willingness, many members of the Alarmed are not actively engaging in advocacy to support climate solutions [16]. Therefore, it would be useful to know what proportion of health professionals are Alarmed and what factors may be hindering their engagement in climate advocacy

To answer these questions, we analyzed data from a Fall 2020 survey of health professionals conducted in a diverse group of more than ten nations [12]. The survey included the “Global Warming's Six Americas Short Survey”—four questions whose answers can be used to reliably categorize respondents into the six audience segments [17]. We determined that 63% of the respondents were members of the Alarmed segment. In comparison, only 26% of the American adult population were members of this segment [14]. This finding suggests that many health professionals around the world are potentially ready to engage in advocacy for health-protecting climate solutions. Therefore, we proceed to the next relevant question: How can Alarmed health professionals be activated to engage in advocacy for climate solutions? In this context, advocacy can take many different forms. First, health professionals can directly lobby their government leaders for stronger climate policies. Second, they can educate the public and other decision-makers to build support for climate solutions. Third, they can lead by example by advocating for the decarbonization of their own hospitals and healthcare facilities.

With this focus in mind, we further analyzed data from Kotcher et al. [12] to examine Alarmed health professionals’ perceptions of the barriers to advocacy, and the resources that might enable it. To that end, we analyzed the responses of Alarmed health professionals and compared them to those of the non-Alarmed group (the other 5 segments combined). We used an overarching principle of behavior activation articulated by Fishbein and Yzer [18] as our guiding framework: “[A]ny behavior is more likely to occur if [a person] has a strong *intention* to perform the behavior, if [they have] the necessary *skills* and *abilities* required to perform the behavior, and if there are no *environmental constraints* preventing behavioral performance” (p.166, emphases ours). Further, in this model, intention was proposed to be influenced by three types of beliefs: beliefs about the risks and benefits of the behavior, how common or socially acceptable the behavior is, and how achievable and effective the behavior is (see Fig. 1 for an illustration).

**Fig. 1:**
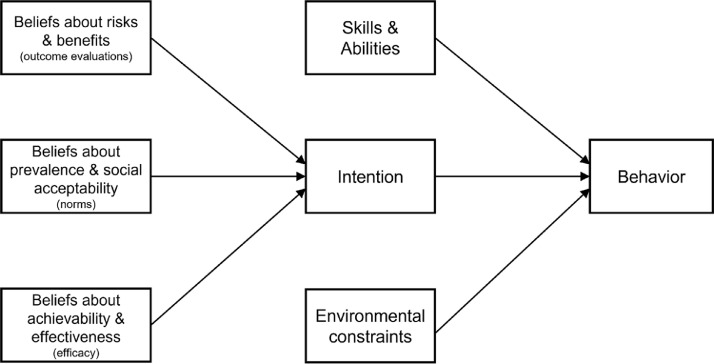
Simplified version of the Integrated Model of Behavioral Prediction (18).

The responses were categorized as appropriate into these three behavioral activation factors: skills and abilities, environmental constraints, and intentions. When present or strong, these factors are resources that facilitate performance of advocacy behavior; when absent or weak, they are barriers or obstacles to advocacy behavior. Tables 1 and 2 show the advocacy barriers and facilitators of Alarmed health professionals categorized by these three factors, some of which belong to more than one factor of behavior activation and were thus classified accordingly.

**Table 1:** Perceived barriers to public advocacy by Alarmed segment (*N* = 2764) and non-Alarmed (*N* = 1655) health professionals.

Behavior Activation Factor		Alarmed	Non-alarmed	Difference
Skills & Abilities	Lack of knowledge	37%	47%	−10
Environmental constraints	Lack of time	55%	51%	+4
	Lack of support from peers	22%	20%	+2
	Too risky professionally/personally	12%	16%	−4
	Too controversial	12%	25%	−13
Intention	Lack of knowledge (perceived achievability)	37%	47%	−10
	Won't make a difference (perceived effectiveness)	29%	34%	−5
	Lack of support from peers (perceived acceptability)	22%	20%	+2
	Too risky professionally/personally (perceived risks)	12%	16%	−4
	Too controversial (perceived risks)	12%	25%	−13

*Note*. The numbers are percentages of respondents who answered ‘a moderate amount’ or ‘a great deal’ to “To what extent do these factors reduce your willingness to communicate with the public about climate change and health?”. Data were collected by Kotcher et al. (12).

**Table 2:** Perceived usefulness of advocacy resources by Alarmed (*N* = 2764) and non-Alarmed (*N* = 1655) health professionals.

Behavior Activation Factor		Alarmed	Non-alarmed	Difference
Skills & Abilities	Continuing professional education	86%	59%	+27
	Guidance to make workplace sustainable	81%	55%	+26
	Communication training	80%	50%	+30
	Patient education materials	75%	46%	+29
Environmental constraints	Policy statement from associations	88%	55%	+33
	Action alerts	82%	47%	+35
	Guidance to make workplace sustainable	81%	55%	+26

*Note*. Numbers are percentages of respondents who answered ‘moderately helpful’ or ‘very helpful’ to “How helpful, if at all, would the following resources be to you?”. Data were collected by Kotcher et al. [12].

In the following, we explore the three key behavioral activation factors and note areas of research and program development necessary for the successful activation of health professionals. For each area, we report the recent findings regarding Alarmed health professionals’ beliefs and perceptions, followed by recommendations for (1) immediate actions for climate and health organizations, and (2) future research directions for social scientists.

## Behavior activation factor: Skills and abilities

Strategies to improve advocacy *skills and abilities* are urgently needed, as they are likely to make the biggest and most immediate impact in activating Alarmed health professionals. Survey responses showed that additional training and educational materials are seen as highly useful by large majorities of Alarmed respondents. In fact, they consistently found these resources to be more helpful for advocacy than non-Alarmed health professionals by a substantial margin (see Table 2). This suggests that many Alarmed health professionals are willing and eager to take actions but lack the necessary tools and skills. Other scholars and practitioners have noted a lack of climate-change-focused content in current medical curricula [19]; our findings suggest that even the most engaged health professionals would benefit from additional training on this topic.

However, such training should not be limited to an overview of the health impacts of climate change. Health professionals also expressed a high degree of interest in developing the communication skills necessary to effectively advocate and in receiving guidance on how to make their workplaces more sustainable. Strengthening resources that help clinicians quickly and efficiently convey the health risks of climate change and the health benefits of climate solutions—such as patient education materials—would also be beneficial. Finally, more efforts are needed to distribute existing resources to aspiring advocates through training webinars, workshops, and advocacy toolkits. Strengthening coordination between advocacy organizations can go a long way in facilitating the dissemination of these readily available resources and avoiding duplication of effort.

In terms of future research, scholars should focus on the development and scaling-up of comprehensive, systematic, and continuous training programs that cultivate the necessary expertise for climate health advocacy. Such an ambitious undertaking requires multidisciplinary scholarship and collaboration. For example, climate and health experts and social scientists can work together to develop accessible, accurate, and compelling messages that health professionals can use to inform a variety of audiences; health educators and interpersonal communication scholars can investigate best practices for educating patients; and political scientists can advise program developers on the most efficient and actionable steps to influence policy makers.

## Behavior activation factor: Environmental constraints

The second cluster of barriers concerns *environmental constraints* to engaging in advocacy actions, including time constraints, lack of leadership and guidance from professional associations, lack of action alerts and peer support, and perceived controversy and riskiness of speaking on the topic. As shown in Table 1, several barriers can be due to either environmental constraints or intention beliefs. For example, barriers related to climate advocacy being too time-consuming, controversial, risky, and unpopular may stem from misperceptions about advocacy that can be simply corrected or from challenges in the professional environment that require deeper institutional change. Fortunately, few Alarmed respondents perceived climate advocacy to be too controversial or risky, though about 1 out of 5 cited a lack of support from their peers. On the other hand, time constraints were the most frequently cited barrier by Alarmed health professionals, along with a great need for more explicit policy and guidance from their professional associations and for timely action alerts.

Addressing these environmental constraints may demand organizational and institutional changes, which presents a catch-22: changing institutional policies and professional practices is required to facilitate advocacy actions, and advocacy actions are required to create these changes. However, this interdependence also means that a positive feedback loop is likely once either factor reaches a critical mass. The challenge, then, is to simultaneously engage potential advocates and push for institutional policy changes until such critical mass is achieved.

Thus, to effectively address these barriers, climate and health organizations need to work on both sides of the equation. First, recruitment efforts should be increased, and requests to take advocacy actions should be made more feasible by providing timely materials and resources, as stated above. Second, training for climate advocacy should include strategies for effective role modeling to help shift professional norms and demonstrate how to balance advocacy work with competing demands. Such preparation can then help Alarmed health professionals advocate for concrete institutional policy changes in their workplaces, such as official policy statements from professional organizations, acknowledging climate advocacy as a part of professional responsibilities and time commitment, and getting leadership to publicly support advocacy actions.

On the research front, organizational psychologists and communication scientists can aid the process by investigating how to best shift institutional policies and professional norms to remove these environmental barriers. Behavioral scientists can work in conjunction with climate and health organizations to optimize the recruitment and activation of future advocates.

As policies and norms around climate advocacy shift, recruitment and activation will become easier; as more health professionals become advocates, institutional policy change will happen more quickly and broadly. These organizational changes are essential if we are to successfully make the necessary impacts on climate issues [20].

## Behavior activation factor: Intention

The last cluster of barriers involves Alarmed health professionals’ *intention* to engage in advocacy behaviors. As mentioned above, Fishbein and Yzer [18] proposed that intention is influenced by three types of beliefs about the characteristics of the behavior: how (1) risky or beneficial; (2) common or socially acceptable; and (3) achievable or effective the behavior is.

Our analyses show that these barriers are the least likely to present challenges to Alarmed health professionals compared to the previous two behavior activation factors. Perceived achievability and effectiveness were most frequently cited with about 1 in 3 respondents thinking they lacked necessary knowledge to advocate or that advocacy won't make a difference. For beliefs about the risks of advocacy or about advocacy being socially unacceptable, the numbers of Alarmed respondents perceiving these as barriers were even lower.

Nevertheless, there is still room for improvement. Strategies to increase advocacy intention should focus on changing health professionals’ perceived lack of effectiveness of advocacy actions, because unlike the other intention barriers, it cannot be addressed by simply improving skills and abilities and removing environmental constraints. For example, publicizing successful advocacy efforts and explicitly connecting positive outcomes to actions can help demonstrate that advocacy actions are indeed effective at bringing about changes.

## Conclusions

In large numbers worldwide, health professionals are Alarmed about climate change and indicate that they are willing to advocate for climate and health solutions. Efforts to activate them should be a high priority for the entire health sector. We recommend that health organizations—including but not limited to professional societies, educational institutions, healthcare and public health systems and agencies, and government and philanthropic funders—prioritize through practice and research: (1) creating and disseminating materials and resources to improve *skills* and *abilities* necessary for climate and health advocacy; (2) promoting institutional policies and professional cultures conducive to advocacy so as to neutralize current *environmental constraints*; and (3) publicizing successful advocacy efforts and outcomes to reduce remaining barriers related to *intention*.

Although our assessment focused on Alarmed professionals as they are most easily activated, it is worth noting that the 37% of non-Alarmed health professionals also have great potential to be activated as climate and health advocates. Compared to the Alarmed group, their biggest obstacle was perceiving climate advocacy to be too controversial. Given the worsening of the climate crisis and the shifting culture in the health sectors, this perception can quickly change, and their participation as advocates will be immensely beneficial.

To succeed, this ambitious agenda will require vigorous and sustained interdisciplinary collaboration between climate and health experts and advocates, social scientists, educators, and other leaders in the health professions. It will also require the necessary funding. Not only is an exceptional effort of this sort *required* to rise to this monumental challenge of our time, making such effort is our *ethical obligation* and *duty of care*.

## Funding

The data collection in Kotcher et al. [12] was supported by funding from WHO. During the conduct of that study, two authors report grants from WHO, and one author reports a grant from the Children's Investment Foundation Fund.

## Declaration of Competing Interest

The authors declare that they have no known competing financial interests or personal relationships that could have appeared to influence the work reported in this paper.
